# Concordance Between the Minimum Data Set Kidney Impairment I1500 Item and eGFR Records in Nursing Homes

**DOI:** 10.1016/j.jamda.2026.106176

**Published:** 2026-04-02

**Authors:** Kamila Radjabova, Nina R. Joyce, Yu-Chia Hsu, Shilo H. McBurney, Arman Oganisian, Andrew R. Zullo

**Affiliations:** a Department of Epidemiology, Brown University School of Public Health, Providence, RI, USA; b Center for Gerontology and Healthcare Research, Brown University School of Public Health, Providence, RI, USA; c Center for Real-World Effectiveness and Safety of Therapeutics, Perelman School of Medicine, University of Pennsylvania, Philadelphia, PA, USA; d Department of Biostatistics, Epidemiology, and Informatics, Perelman School of Medicine, University of Pennsylvania, Philadelphia, PA, USA; e Department of Biostatistics, Brown University School of Public Health, Providence, RI, USA; f Transformative Health Systems Research to Improve Veteran Equity and Independence Center of Innovation (THRIVE COIN), Providence Veterans Affairs Medical Center, Providence, RI, USA; g Department of Pharmacy, Brown University Health, Rhode Island Hospital, Providence, Rhode Island, USA

**Keywords:** Glomerular filtration rate, kidney diseases, Minimum Data Set, nursing homes, validation study

## Abstract

**Objectives:**

To evaluate the concordance between the Minimum Data Set (MDS) version 3.0 I1500 item and estimated glomerular filtration rate (eGFR) laboratory values for identifying kidney impairment among nursing home (NH) residents.

**Design:**

A retrospective cohort study was conducted using data from 8 multistate, multifacility NH chains from January 1, 2018, to July 1, 2022. MDS 3.0 assessments with a complete I1500 item were linked to eGFR values measured on the assessment reference date or within 7 days prior.

**Setting and Participants:**

The study included NH residents with at least 1 MDS 3.0 assessment linked to a matching eGFR within a 7-day look-back window.

**Methods:**

Concordance between the 2 measures was evaluated using sensitivity, specificity, positive predictive value, negative predictive value, and Cohen’s kappa statistics, overall and stratified by pandemic periods (pre-pandemic, early pandemic, and late pandemic). We used an eGFR threshold of <60 mL/min/1.73 m^2^ for the primary analysis and <45 for the secondary analysis.

**Results:**

The study included 454,436 MDS assessments from 225,557 NH residents. Using an eGFR threshold of <60, agreement with the MDS I1500 item was 60.3% (kappa = 0.19), with 25.8% sensitivity and 92.3% specificity. At the <45 threshold, agreement rose to 71.8% (kappa = 0.22), with 29.9% sensitivity and 89.2% specificity.

**Conclusions and Implications:**

The MDS 3.0 I1500 measure demonstrated fair agreement, low sensitivity, and high specificity for identifying kidney impairment compared with eGFR values. Cross-referencing federally mandated MDS data with eGFR data could improve kidney impairment identification and care planning in NHs.

The Minimum Data Set (MDS) for resident assessment has been federally required in all US nursing homes (NHs) since 1990.^1,2^ Originally designed for care planning, the MDS has evolved into a multifunctional tool for quality measurement, reimbursement, and research, making the validity of its condition indicators important for contemporary NH research and quality monitoring.^2,3^ The latest version, 3.0, was implemented on October 10, 2010.^1^

The MDS includes the measure I1500 for “renal insufficiency, renal failure, or end-stage renal disease (ESRD)” in its “Active Diagnoses” section.^1^ Although phrased clinically, I1500 functions as the MDS indicator of kidney impairment, which affects nearly half of long-term care NH residents.^4,5^ Despite rising overall diagnosis rates, kidney impairment remains underdiagnosed, leading to potential mismeasurement in NHs.^5^ Although clinicians typically manage conditions like kidney impairment based on laboratory values, researchers, quality monitoring programs, and policy initiatives often rely on MDS data to identify residents with particular conditions measured in Section I of the MDS. For example, researchers might use the MDS I1500 measure to identify NH residents with kidney impairment in observational studies and evaluate whether medications were appropriately dose-adjusted for renal function.^6^

Kidney impairment is particularly concerning in NH residents, who often have multiple comorbidities and are subject to polypharmacy.^7^ The COVID-19 pandemic has potentially exacerbated these issues, both by contributing to increased kidney impairment and by straining NH resources, potentially affecting the accuracy of MDS documentation.^8^ COVID-19 has been associated with direct kidney injury, particularly among older adults, leading to an increased incidence of acute kidney injury (AKI) and long-term kidney impairment.^9^ Some NHs even implemented COVID-19 kidney function monitoring protocols to help guide infection management. Simultaneously, NHs experienced significant resource constraints, including staffing shortages and increased clinical burden, which may have impacted the accuracy and consistency of MDS documentation.^8^ These challenges may have reduced the time and clinical detail available for accurate MDS documentation, particularly at admission or during periods of rapid resident turnover. Examining potential discrepancies between MDS I1500 and laboratory-based measures of kidney function across pandemic periods may provide insights into the extent of these disruptions. Although acute COVID-19 disruptions have waned, stratification on pandemic periods provides a natural opportunity to assess MDS documentation under operational strain and helps clarify whether concordance is a time-limited pandemic artifact or a persistent measurement issue.

One key measure of kidney function is the glomerular filtration rate (GFR). Estimated GFR (eGFR), which is based on serum creatinine levels, is the most commonly used measure of GFR in clinical practice and research.^10,11^ Although exogenous filtration markers like inulin provide a more accurate measure of GFR than eGFR, they are costly and impractical for routine use, particularly in NHs.^12^ In NH settings, eGFR values are often available through laboratory panels and are documented in electronic health records (EHRs), where they frequently inform medication dosing and clinical decision making, making eGFR a pragmatic reference standard measure of kidney function.^10,11^

This study aimed to assess the concordance between the MDS 3.0 I1500 and eGFR measures for identifying kidney impairment, both overall and stratified by pandemic time period. Our goal was to estimate the sensitivity, specificity, positive predictive value (PPV), and negative predictive value (NPV) of the MDS I1500 measure compared with eGFR for identifying kidney impairment. We hypothesized that the MDS I1500 and eGFR measures of kidney impairment would often disagree, with greater discrepancies during the pandemic due to both shifts in kidney impairment prevalence among NH residents and potential disruptions in MDS documentation practices.

## Methods

### Data Sources

Our team partnered with NH chains using a common EHR system (PointClickCare). The chains included more than 1100 facilities across the contiguous United States, with approximately 75,100 beds. The resident population consisted of individuals receiving both post-acute and long-term NH care. The NH EHR records included resident demographics (eg, age, sex, race/ethnicity), laboratory data, vital signs (eg, blood pressure, temperature), diagnoses, and MDS assessments. The federally mandated MDS assessments occur at admission and at least quarterly thereafter. Nursing staff conduct the MDS assessments using various methods, including EHR documentation, staff consultations, and direct interviews of residents or their legally authorized representatives.^1^

### Study Design

We conducted a retrospective observational cohort study using data from 8 of the NH chains from January 1, 2018 to July 1, 2022. Data from July 1, 2022 were the most recent available and approved for use. The NH EHR data that documented eGFR and MDS 3.0 assessments were linked for analysis, such that there were 2 sources of kidney impairment information for all observations in the analytic sample. The study was approved by the Brown University Institutional Review Board.

### Study Sample

**Fig. 1.**
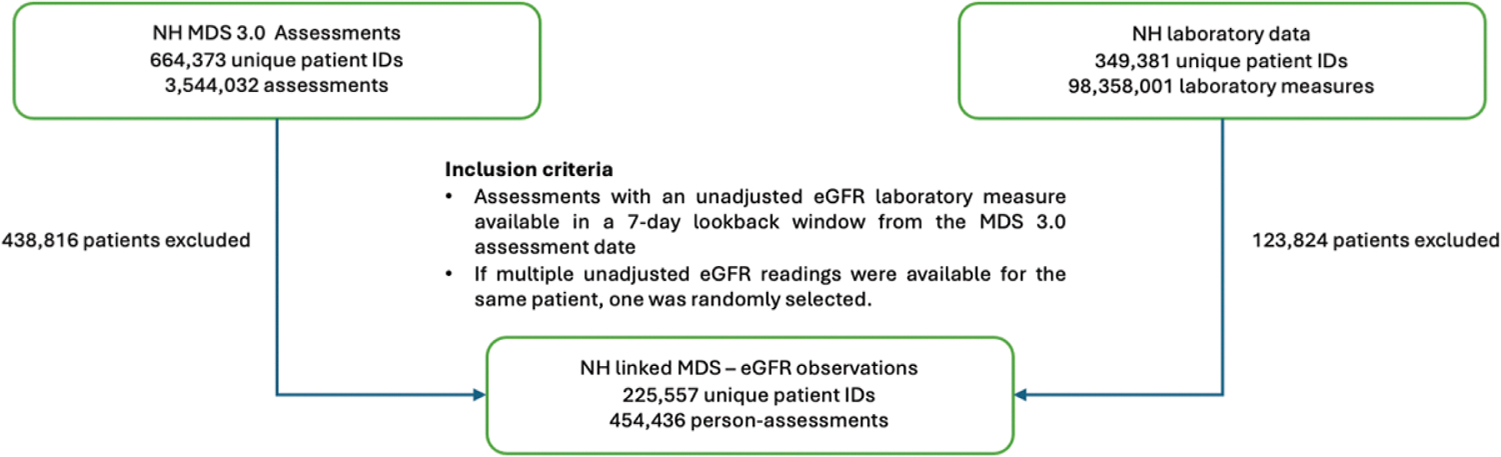
Flowchart of sample selection. ID, identifier; Unadjusted, unadjusted for race.

The study sample consisted of NH residents who had at least 1 MDS 3.0 assessment paired with an eGFR value within a 7-day window before and including the MDS assessment date during the study period (Figure 1). A 7-day window was used because, per the MDS 3.0 Resident Assessment Instrument Manual, Section I (“Active Diagnoses”) items are coded based on whether the condition was active during the 7-day look-back period ending on the assessment reference date, after first confirming documentation of the diagnosis within the prior 60 days. Thus, the I1500 measure identifies an active diagnosis of kidney impairment within a 7-day look-back from the date of assessment.^1^ If multiple eGFR values were eligible for the same person identifier, assessment date, and assessment type, one value was randomly sampled for inclusion in the analytic sample. The unit of analysis was therefore a person-assessment, which was a unique pair of MDS I1500 and eGFR values for a particular person at a particular time. Multiple person-assessments could be contributed to the analytic sample by a unique person over time if they had multiple MDS assessments documenting the I1500 measure with corresponding eGFR values, which respected the longitudinal nature of renal function monitoring in NHs. We did not limit the NH population by age, insurance payer, or any other eligibility criterion.

### MDS 3.0 I1500 Kidney Impairment Measure

The primary variable of interest was the I1500 item for “renal insufficiency, renal failure, or ESRD” (“Yes”/“No”), which is coded based on observed signs and symptoms of kidney impairment.^1,13^ Per the MDS 3.0 RAI manual, diagnoses in Section I are coded as active only if (1) the diagnosis is documented within the prior 60 days by a physician or physician-equivalent clinician and (2) the diagnosis has a direct relationship to the resident’s current functional status, cognitive status, mood or behavior status, medical treatments, nursing monitoring, or risk of death during the 7-day look-back period.^1^ Conditions that are resolved, no longer affect the resident’s current status, or do not drive the care plan during the look-back period are not coded as active.^1^

### eGFR Laboratory Measure

eGFR values (mL/min/1.73 m^2^) within a 7-day look-back window from an MDS assessment were extracted from EHRs. eGFR can be categorized to organize kidney impairment into 5 stages of severity that map to chronic kidney disease (CKD) definitions^10,14^: stage 1 (eGFR >90), normal function with possible kidney damage; stage 2 (eGFR 60–89), mild decrease in function with damage; stage 3 (eGFR 30–59), moderate decrease, may require treatment; stage 4 (eGFR 15–29), severe decrease, preparation for dialysis needed; and stage 5 (eGFR <15), kidney failure, requiring dialysis or transplant.^14^ We used eGFR values as reported by the laboratories serving the NHs. The description of the reported eGFR value rarely specified whether a particular equation was used [eg, Chronic Kidney Disease Epidemiology Collaboration (CKD-EPI) 2009]. Race adjustments were inconsistently documented in the descriptions. When both race-adjusted and unadjusted values were available for the same person identifier, assessment date, and assessment type, we retained the unadjusted value and if there were multiple unadjusted values, we then randomly sampled one of them. We did not recalculate eGFR values from serum creatinine.

A new variable was created to define kidney impairment as a “Yes” for eGFR <60 in the primary analysis, and for eGFR <45 in the secondary analysis. It is important to note that the MDS manual does not define a specific threshold of kidney impairment for the I1500 variable, and the kidney impairment indication was based on clinical judgment rather than a fixed eGFR cutoff. An eGFR <60 (CKD stage 3 or worse) is both the threshold most frequently used in prior research and the threshold at which dose adjustment is recommended for many commonly prescribed drugs whose excretion is affected by kidney impairment.^15^

We defined a more restrictive alternative threshold of eGFR at <45 because that threshold specifically corresponds to CKD stage 3b or worse. The alternative threshold has also been used in large validation studies conducted outside of NH populations.^15^

Including both eGFR thresholds allowed us to evaluate how MDS I1500 performs for (1) a broader population that may require monitoring (<60) and (2) the subset of the population at greater risk for progression to ESRD or adverse effects of medications cleared by the kidneys (<45).

### Demographic and Pandemic Covariates

Demographic covariates used to describe the study population included age, sex, race/ethnicity, and marital status. Age was a continuous variable, ranging from 16 to 105 years. Residents younger than 65 years were included to improve generalizability of the study findings to the entire NH population regardless of age. Assessment type (discharge, admission, status change) was evaluated by pandemic time (prepandemic: January 1, 2018, to February 29, 2020; early pandemic: March 1, 2020, to December 31, 2021; late pandemic: January 1 to July 1, 2022).

### Statistical Analyses

Descriptive statistics were employed to summarize the demographic characteristics of the study population and the distribution of key variables. Resident clinical and administrative characteristics also were summarized across pandemic periods to contextualize temporal differences in concordance statistics.

To evaluate the concordance between the MDS 3.0 I1500 measure (“Yes”/“No”) and an eGFR value of <60 (“Yes”/“No”), we calculated concordance statistics, including percent agreement, Cohen’s kappa statistic, sensitivity, specificity, NPV, PPV, false negative rate, and false positive rate. Because multiple MDS I1500-eGFR pairs could be contributed by the same resident, all concordance statistics were bootstrapped at the resident level with 1000 replicates to account for within-subject correlation and provide correct standard errors. Wald-style 95% confidence intervals (CIs) were constructed for all concordance statistics using the resident-level bootstrap standard error.

Analyses were stratified by pandemic period to examine potential temporal changes in MDS 3.0 kidney impairment identification accuracy. Separate metrics (eg, kappa) were computed within each period.

All statistical analyses were performed using SAS version 9.4 (SAS Institute).^16^

## Results

### Study Population

A total of 454,436 MDS 3.0 person-assessments from 225,557 eligible NH residents (mean ± SD age: 74.8 ± 12.8 years; 54.3% female; 70.8% White) were matched to eGFR values within 7 days of the assessment date. Residents contributed a mean of 2.01 assessments (SD: 1.76; range: 1–45). Kidney impairment was identified in 16.4% of assessments using the MDS I1500 item, 48.2% using the eGFR <60 threshold, and 29.3% using the <45 threshold. Most assessments were admission (46.4%), followed by discharge and status change (Tables 1 and 2).

### Primary Analysis (<60 mL/min/1.73 m^2^ eGFR Threshold)

Overall agreement between the MDS 3.0 kidney impairment measure and the eGFR <60 indicator was 60.3% (95%CI 59.8–60.7), with a kappa statistic of 0.19 (0.18–0.20) (Table 3). Agreement stratified by pandemic period was 59.5% (59.1–60.0) before the pandemic, 61.0% (60.5–61.5) in the early pandemic, and 60.8% (60.2–61.3) in the late pandemic.

The overall sensitivity and specificity of the MDS I1500 item were 25.8% (24.6–27.0) and 92.3% (92.0–92.5), respectively (Table 4), when comparing to eGFR as the reference standard. Sensitivity by pandemic period was 20.9% (19.8–22.1) before the pandemic, 30.6% (29.4–31.8) in the early pandemic, and 30.2% (29.1–31.3) in the late pandemic. Specificity by pandemic period was 94.2% (94.0–94.5) before the pandemic, 90.2% (89.9–90.5) in the early pandemic, and 90.5% (90.1–90.9) in the late pandemic.

The overall PPV and NPV were 75.7% (75.2–76.1) and 57.2% (56.8–57.7), respectively (Table 4). PPV by pandemic period was 76.6% (75.9–77.2) before the pandemic, 74.9% (74.3–75.5) in the early pandemic, and 75.6% (74.6–76.6) in the late pandemic periods. NPV by pandemic period was 57.0% (56.6–57.4) before the pandemic, 57.1% (56.6–57.6) in the early pandemic, and 57.1% (56.6–57.6) in the late pandemic periods.

### Secondary Analysis (<45 mL/min/1.73 m^2^ eGFR Threshold)

Overall agreement between the MDS 3.0 kidney impairment measure and the eGFR <45 indicator was 71.8% (71.6–72.1), with a kappa statistic of 0.22 (0.21–0.23) (Supplementary Table 1). The overall sensitivity and specificity of the MDS I1500 measure were 29.9% (28.4–31.5) and 89.2% (88.8–89.5), respectively. Sensitivity by pandemic period was 24.6% (23.2–26.1) before the pandemic, 35.2% (33.7–36.7) in the early pandemic, and 34.7% (33.3–36.1) in the late pandemic (Supplementary Table 2).

## Discussion

Our cohort study aimed to quantify the agreement, sensitivity, specificity, PPV, and NPV comparing the MDS 3.0 I1500 and eGFR measures of kidney impairment overall and across pandemic periods. The key finding of our study was that the sensitivity of the MDS 3.0 I1500 measure was poor for identifying kidney impairment when compared with eGFR as a pragmatic reference standard. Across all periods, the MDS I1500 identified only about 1 in 4 individuals who were identified as having kidney impairment based on the eGFR values. This level of discordance suggests that the I1500 item may substantially underascertain kidney impairment when used as a standalone indicator in administrative datasets. At the same time, because I1500 is intended to capture an active diagnosis affecting current status and care planning, some instances in which eGFR is below threshold but I1500 is unchecked may reflect transient or reversible kidney impairment (eg, dehydration- or illness-associated AKI, short-term medication effects) rather than CKD (ie, the MDS I1500 and eGFR are related, but not identical constructs). Although the misclassification of the I1500 measure is unlikely to directly alter clinical decisions because NH clinicians rely on eGFR values, it may introduce bias into research or quality metrics that depend on the MDS to identify residents with kidney impairment. Researchers with access to both eGFR and MDS data should prioritize eGFR values over the MDS when measuring kidney impairment in NH residents. Our empirical sensitivity and specificity estimates, overall and stratified by pandemic period, and 2 eGFR thresholds, also provide practical inputs for quantitative bias analyses; investigators who only have access to MDS I1500 in their datasets can use these parameters in probabilistic sensitivity analyses to adjust for misclassification.

These findings are consistent with prior inferences about the accuracy of MDS 3.0 documentation. Studies by Wei et al^17,18^ identified similar performance of MDS items for depression, pain, and behavioral symptoms, which exhibited low concordance with International Classification of Diseases, Ninth/10^th^ Revision diagnoses codes in Medicare claims data. The MDS I1500 measure was more effective at ruling out kidney impairment than confirming its presence (ie, was highly specific), reflecting a broader theme in the evidence on the validity of the MDS items, whereby items are usually better at identifying the absence rather than the presence of conditions.^17,18^

Across pandemic periods, I1500 sensitivity increased while specificity declined, with a modest increase in overall agreement and kappa. One shift in documentation practices that may have contributed to increased sensitivity is that Section I diagnoses may have been more likely to be copied forward in time from prior MDS assessments into subsequent assessments rather than be dynamically updated, potentially to efficiently use staff time during the pandemic. Similarly, NH staff may have been more likely to copy forward hospital discharge diagnoses into the EHR problem list and MDS Section I. Clinicians also increased renal laboratory testing for COVID-19 management, hydration assessment, and medication monitoring, which made transient abnormalities in kidney function more likely to appear in clinical documentation. The greater availability of eGFR information could have contributed to higher I1500 sensitivity but lower specificity, as some newly documented renal impairments reflected acute rather than CKD.

Following the onset of the COVID-19 pandemic, we observed a notable shift in the distribution of assessment types: admission assessments declined from 52.3% pre-pandemic to 40.7% in the late pandemic period, while status and discharge assessments increased (Table 2). Even prior to COVID-19, structural barriers such as inadequate staffing, high turnover, limited training, and time pressures were identified as impediments to accurate assessment in NHs.^19^ The pandemic likely exacerbated these issues, as has been documented in more recent literature describing widespread staffing shortages and operational strain in long-term care settings.^20,21^ NH admissions declined substantially during the pandemic, driven by reduced bed availability, infection control concerns, and shifts toward homebased care alternatives when possible.^22^ These systemic strains may have further hindered the ability of NHs to complete admission assessments in line with pre-pandemic standards, contributing to the observed shift in assessment composition. Although our analysis did not stratify concordance statistics by assessment type, this shift may have indirectly affected MDS documentation practices.^8,19–21^

When using the <45 threshold, the MDS kidney impairment measure demonstrated slightly higher sensitivity (29.9%) but lower specificity (89.2%) compared with the <60 threshold, indicating modest improvement in detecting more severe kidney impairment. However, the MDS’s continued underdetection of kidney impairment at this threshold highlights the potential danger in relying on the MDS I1500 measure alone to identify those residents most in need of kidney-specific care when conducting research or quality measurement. This is particularly concerning given that individuals with eGFR values of <45 are at even higher risk for medication-related adverse effects. They require more substantial dose adjustments than individuals with eGFR values <60 to account for their decreased ability to eliminate many medications and potential for higher than intended blood serum concentrations.^23^ It is important to note that the <60 threshold more frequently captures transient or reversible changes in renal function due to acute illness, dehydration, or medication effects than the <45 threshold for kidney impairment, which likely explains the lower overall agreement with the MDS I1500 measure at the <60 threshold. This distinction is also important for interpretation: some observations categorized as “false negatives” at the <60 threshold may represent temporary eGFR declines documented during acute illness or dehydration that appropriately would not be coded as an active diagnosis on I1500 if they resolved and did not meaningfully influence the resident’s current status or care plan during the MDS look-back.

### Limitations

A primary potential limitation of this study is the selection of eGFR thresholds. eGFR can be influenced by factors such as creatinine assay methods, hydration status, and muscle mass, which may introduce variability and affect the reliability of these thresholds.^10^ In addition, our dataset included a wide variety of eGFR test names and calculation approaches, reflecting differences in laboratory systems and clinical workflows. This heterogeneity may have introduced inconsistencies in measurement or interpretation, increasing the risk of misclassification. It is also important to note that eGFR might be an excellent reference standard, but it is not a gold standard like inulin or other exogenous filtration markers. Moreover, because we linked each assessment to an eGFR value within a 7-day window, some paired eGFR values may have reflected acute and reversible physiologic changes (eg, dehydration-related AKI) rather than stable CKD, which could inflate discordance when compared with an “active diagnosis” measure such as I1500. Discordance can therefore arise even when both the I1500 and eGFR measures are accurate. Although the study stratified by pandemic periods to assess disruptions, unmeasured factors such as staff training variability, institutional policies, and access to laboratory results may have influenced documentation accuracy and were not measurable in the data. Furthermore, kidney function monitoring is often driven by medication management (eg, for diuretics, angiotensin-converting enzyme inhibitors, antibiotics), acute illness or infection, dehydration, and admission to the NH, but we did not have direct measures of the reasons for kidney function assessment in our data and could not examine the concordance between eGFR and the MDS I1500 stratified by or taking into account of those reasons.

## Conclusions and Implications

This study identified substantial discrepancies between the MDS 3.0 I1500 item and eGFR, with sensitivity of the MDS item persistently low across several analyses and approximately 70% of NH residents with eGFR-identified kidney impairment unrecorded in MDS assessments.

The documented discrepancies have important implications for NH research and quality measurement because reliance on the MDS I1500 item alone may substantially underascertain kidney impairment. To improve the accuracy of kidney impairment documentation, we recommend integrating eGFR measures directly into the workflow for completing the federally mandated MDS assessments. Automating this process would enhance the accuracy and consistency of documentation, thereby potentially improving research and quality improvement initiatives.^22–26^ The findings also highlight the need for policy efforts focused on strengthening long-term care technological infrastructure, staff training, and documentation practices in NHs.^21–26^

Future researchers should consider estimating the effects of MDS-eGFR discrepancies on clinical outcomes, such as medication adverse effects, hospitalizations, and mortality. Such studies could inform quality improvement and policy efforts focused on improving the accuracy of MDS documentation by demonstrating the consequences of discrepant documentation in NHs.

## Supplementary Material

Supplement

Supplementary data related to this article can be found online at https://doi.org/10.1016/j.jamda.2026.106176.

## Figures and Tables

**Table 1:** Characteristics of Eligible NH Residents, January 1, 2018, to July 1, 2022 (N = 454,436 Person-Assessments)

Characteristic	N = 454,436 Person-Assessments*	%^†^

Age, y, mean (SD)^‡^	74.8 (12.8)	
Sex		
Female	246,891	54.3
Male	207,545	45.7
Race/Ethnicity		
American Indian/Alaska Native	1202	0.3
Asian	11,756	2.6
Black/African American	88,495	19.5
Hispanic/Latino	13,219	2.9
Hawaiian/Other Pacific Islander	2165	0.5
White	321,727	70.8
Unknown/Missing	15,872	3.5
Marital status		
Divorced	69,821	15.4
Married	137,754	30.3
Never married	10,650	2.3
Separated	816	0.2
Widowed	128,470	28.3
Unknown	106,925	23.5
MDS 3.0 I1500 measure		
Yes	74,632	16.4
No	379,804	83.6
eGFR <45 mL/min/1.73 m^2^		
Yes	133,298	29.3
No	321,138	70.7
eGFR <60 mL/min/1.73 m^2^		
Yes	218,948	48.2
No	235,488	51.8
Pandemic period		
Before pandemic	228,489	50.3
Early pandemic	179,432	39.5
Late pandemic	46,515	10.2
Type of assessment		
Discharge	161,042	35.4
Admission	211,062	46.4
Status change	82,332	18.1
NH chain^§^		
1	21,739	4.8
2	24,552	5.4
3	72,773	16.0
4	248,579	54.7
5	16,827	3.7
6	48,510	10.7
7	1915	0.4
8	19,541	4.3

* Number of unique residents was 225,557.

† Percentages may not fully sum to 100 due to dummy-coded variables and rounding.

‡ Age is based on a randomly selected MDS assessment per resident.

§ Each NH chain was assigned a number to maintain confidentiality and comply with the data use agreement.

**Table 2:** Distribution of Kidney Impairment Indicators and Assessment Type by Pandemic Period, January 1, 2018-July 1, 2022 (N = 454,436 Person-Assessments)*

Characteristic	Pre-pandemic (n = 228,489)	Early Pandemic (n = 179,432)	Late Pandemic (n = 46,515)
	n (%)^†^	n (%)^†^	n (%)^†^

MDS I1500 = “Yes”	29,562 (12.9)	35,886 (20.0)	9184 (19.7)
eGFR <60 mL/min/1.73 m^2^ = “Yes”	108,209 (47.4)	87,784 (48.9)	22,955 (49.4)
eGFR <45 mL/min/1.73 m^2^ = “Yes”	65,541 (28.7)	53,810 (30.0)	13,947 (30.0)
Type of assessment			
Discharge	73,881 (32.3)	68,969 (38.4)	18,192 (39.1)
Admission	119,545 (52.3)	72,586 (40.5)	18,931 (40.7)
Status change	35,063 (15.4)	37,877 (21.1)	9392 (20.2)

* Number of unique residents was 225,557.

† Percentages represent the proportion of assessments with the study characteristic out of all assessments conducted during the specified period.

**Table 3:** Concordance Between Kidney Impairment Assessed in MDS 3.0 and Documented in eGFR Laboratory Values (<60 mL/min/1.73 m^2^) During the 7 Days Before an Eligible MDS 3.0 Assessment Date and Stratified Concordance Statistics on Pandemic Time, January 1, 2018, to July 1, 2022 (N = 454,436 Person-Assessments)*

Pandemic Period	n	eGFR <60 and MDS = Yes	eGFR <60 and MDS = No	eGFR ≥60 and MDS = Yes	eGFR ≥60 and MDS = No	Percent Agreement	Kappa
					
		n (%)	n (%)	n (%)	n (%)	(95% CI)	(95% CI)

All observations	454,436	56,463 (12.4)	162,485 (35.8)	18,169 (4.00)	217,319 (47.8)	60.3 (59.8–60.7)	0.19 (0.18–0.20)
Before pandemic	228,489	22,642 (9.91)	85,567 (37.5)	6920 (3.03)	113,360 (49.6)	59.5 (59.1–60.0)	0.16 (0.15–0.17)
Early pandemic	179,432	26,880 (15.0)	60,904 (33.9)	9006 (5.02)	82,642 (46.1)	61.0 (60.5–61.5)	0.21 (0.20–0.22)
Late pandemic	46,515	6941 (14.9)	16,014 (34.4)	2243 (4.82)	21,317 (45.8)	60.8 (60.2–61.3)	0.21 (0.20–0.22)

* Number of unique residents was 225,557.

**Table 4:** PPVs and NPVs, Sensitivity, Specificity, and False Positive/Negative Rates of MDS 3.0 Kidney Impairment Measure Compared With eGFR Kidney Impairment Threshold (<60 mL/min/1.73 m^2^)) in Study Sample, January 1, 2018, to July 1, 2022 (N = 454,436 Person-Assessments)*

	n	PPV	NPV	Sensitivity	Specificity	False Negative Rate	False Positive Rate
		(95% CI)	(95% CI)	(95% CI)	(95% CI)	(95% CI)	(95% CI)

Overall	454,436	75.7 (75.2–76.1)	57.2 (56.8–57.7)	25.8 (24.6–27.0)	92.3 (92.0–92.5)	74.2 (73.0–75.4)	7.72 (7.48–7.95)
Before pandemic	228,489	76.6 (75.9–77.2)	57.0 (56.6–57.4)	20.9 (19.8–22.1)	94.2 (94.0–94.5)	79.1 (77.9–80.2)	5.75 (5.52–5.99)
Early pandemic	179,432	74.9 (74.3–75.5)	57.1 (56.6–57.6)	30.6 (29.4–31.8)	90.2 (89.9–90.5)	69.4 (68.2–70.6)	9.83 (9.54–10.1)
Late pandemic	46,515	75.6 (74.6–76.6)	57.1 (56.6–57.6)	30.2 (29.1–31.3)	90.5 (90.1–90.9)	69.8 (68.7–70.9)	9.52 (9.13–9.91)

* Number of unique residents was 225,557.
