# Supplementary material for: Concordance Between the Minimum Data Set Kidney Impairment I1500 Item and eGFR Records in Nursing Homes
Source: J Am Med Dir Assoc. Author manuscript; Available in PMC 2026 May 12. (PMC13158888; doi:10.1016/j.jamda.2026.106176)
Supplement: Supplement [file NIHMS2166457-supplement-Supplement.docx]

**SUPPLEMENTARY MATERIALS**

**Title**: Concordance Between the Minimum Data Set Kidney Impairment I1500 Item and eGFR Records in Nursing Homes

**Supplementary Table S1.** Concordance Between Kidney Impairment Assessed in MDS 3.0 and Documented in eGFR Laboratory Values (<45 mL/min/1.73m^2^) During the 7 Days Before an Eligible MDS 3.0 Assessment Date and Concordance Statistics Stratified on Pandemic Time, January 1, 2018 to July 1, 2022.

**Supplementary Table S2.** Positive and Negative Predictive Values, Sensitivity, Specificity, and False Positive/Negative Rates of MDS 3.0 Kidney Impairment Measure Compared to eGFR Kidney Impairment Threshold (<45mL/min/1.73m^2^) in Study Sample, January 1, 2018 to July 1, 2022.

| **Supplementary Table S1.** Concordance Between Kidney Impairment Assessed in MDS 3.0 and Documented in eGFR Laboratory Values (<45 mL/min/1.73m^2^) During the 7 Days Before an Eligible MDS 3.0 Assessment Date and Concordance Statistics Stratified on Pandemic Time, January 1, 2018 to July 1, 2022 (N = 454,436 person-assessments).* | | | | | | | |
| --- | --- | --- | --- | --- | --- | --- | --- |
| **Pandemic Period** | **N** | **eGFR <45 & MDS = Yes** | **eGFR <45 & MDS = No** | **eGFR ≥45 & MDS = Yes** | **eGFR ≥45 & MDS = No** | **Percent Agreement** | **Kappa** |
|  |  | **n (%)** | **n (%)** | **n (%)** | **n (%)** | **(95% CI)** | **(95% CI)** |
| **All observations** | 454,436 | 39,911  (8.8) | 93,387  (20.6) | 34,721  (7.6) | 286,417  (63.0) | 71.8  (71.6–72.1) | 0.22 (0.21–0.23) |
| **Before pandemic** | 228,489 | 16,132  (7.1) | 49,409  (21.6) | 13,430 (5.9) | 149,518 (65.4) | 72.5  (72.2–72.8) | 0.20 (0.18–0.21) |
| **Early pandemic** | 179,432 | 18,935 (10.6) | 34,875 (19.4) | 16,951  (9.5) | 108,671  (60.5) | 71.1  (70.9–71.4) | 0.24 (0.23–0.25) |
| **Late pandemic** | 46,515 | 4,844  (10.4) | 9,103  (19.6) | 4,340 (9.3) | 28,228 (60.7) | 71.1  (70.7–71.5) | 0.24  (0.23–0.25) |
| Abbreviations: CI, confidence interval, MDS, Minimum Data Set, eGFR, estimated glomerular filtration rate. *Number of unique residents was 225,557. | | | | | | | |

| **Supplementary Table S2.** Positive and Negative Predictive Values, Sensitivity, Specificity, and False Positive/Negative Rates of MDS 3.0 Kidney Impairment Measure Compared to eGFR Kidney Impairment Threshold (<45mL/min/1.73m^2^) in Study Sample, January 1, 2018 to July 1, 2022 (N = 454,436 person-assessments).* | | | | | | | |
| --- | --- | --- | --- | --- | --- | --- | --- |
|  | **N** | **Positive Predictive Value** | **Negative Predictive Value** | **Sensitivity** | **Specificity** | **False Negative Rate** | **False Positive Rate** |
|  |  | **(95% CI)** | **(95% CI)** | **(95% CI)** | **(95% CI)** | **(95% CI)** | **(95% CI)** |
| **Overall** | 454,436 | 53.5  (53.0 – 54.0) | 75.4  (75.0 – 75.8) | 29.9  (28.4 – 31.5) | 89.2  (88.8 – 89.5) | 70.1  (68.5 – 71.6) | 10.8  (10.5 – 11.2) |
| **Before Pandemic** | 228,489 | 54.6  (53.8 – 55.3) | 75.2  (74.8 – 75.5) | 24.6  (23.2 – 26.1) | 91.8  (91.4 – 92.1) | 75.4  (73.9 – 76.8) | 8.24  (7.89 – 8.59) |
| **Early Pandemic** | 179,432 | 52.8  (52.1 – 53.4) | 75.7  (75.3 – 76.1) | 35.2  (33.7 – 36.7) | 86.5  (86.1 – 86.9) | 64.8  (63.3 – 66.3) | 13.5  (13.1 – 13.9) |
| **Late Pandemic** | 46,515 | 52.7  (51.7 – 53.8) | 75.6  (75.1 – 76.1) | 34.7  (33.3 – 36.1) | 86.7  (86.2 – 87.1) | 65.3  (63.9 – 66.7) | 13.3  (12.9 – 13.8) |
| *Number of unique residents was 225,557. | | | | | | | |
